# Heat-shock induces rapid resorption of primary cilia

**DOI:** 10.1186/2046-2530-1-S1-P52

**Published:** 2012-11-16

**Authors:** CL Thompson, NV Prodromou, DP Osborn, R Ashworth, MM Knight, PL Beales, JP Chapple

**Affiliations:** 1 Queen Mary University of London, UK; 2 Barts and the London School of Medicine and Dentistry, UK; 3 UCL Institute of Child Health, UK

## 

Primary cilia are involved in important developmental and disease pathways, such as the regulation of neurogenesis and tumorigenesis. They function as sensory antennae and are essential in the regulation of key extracellular signalling systems. In this study we investigate the effects of cell stress on primary cilia. Exposure of mammalian cells in vitro, and zebrafish cells in vivo, to elevated temperature resulted in the rapid loss of cilia by resorption. In mammalian cells cilia loss correlated with a reduction in ligand dependent hedgehog signalling. Heat shock dependent loss of cilia was decreased in cells where histone deacetylases (HDACs) were inhibited, suggesting resorption is mediated by HDAC6 which localises to ciliary axonemes. The rate of cilia resorption was reduced in thermotolerant cells. This implies a role for molecular chaperones in primary cilia maintenance. The cytosolic chaperone Hsp90 localised to the ciliary axoneme and its inhibition resulted in cilia loss. In the cytoplasm of unstressed cells Hsp90 is known to exist in a complex with HDAC6. Immediately after heat shock Hsp90 levels were reduced in remaining ciliary axonemes. We hypothesise that cilia resorption in response to heat shock is regulated by the disassembly of an HDAC6/Hsp90 complex and would serve to attenuate cilia mediated signalling pathways and reduce the translational load on the cell in times of stress.

